# Intelligence Quotient (IQ) in school-aged preterm infants: A systematic review

**DOI:** 10.3389/fpsyg.2023.1216825

**Published:** 2023-07-25

**Authors:** Laura Lacalle, Melissa Liher Martínez-Shaw, Yolanda Marín, Yolanda Sánchez-Sandoval

**Affiliations:** ^1^Department of Psychology, University of Cádiz, Cádiz, Spain; ^2^Instituto de Investigación e Innovación Biomédica de Cádiz (INiBICA), Cádiz, Spain

**Keywords:** preterm birth, prematurity, school age, cognitive development, intelligence quotient, IQ

## Abstract

Preterm birth (before 37 weeks of gestational age) is associated with certain risks to child development. The aim of this systematic review was to summarize available and updated empirical evidence on prematurity as a risk factor for cognitive development in school age. Thus, we attempted to identify similarities and differences with the full-term population and to point out possible risk or protective factors among the biological, psychosocial and family variables. The conceptualization and methodology of this review followed the PRISMA recommendations. The search was carried out in Web of Science, Scopus, PsycInfo, and Dialnet databases, in May 2022. The search was limited to journal articles, published between 2012 and 2022, in English and Spanish. Research articles selected were those focused on the intelligence quotient (IQ) of preterm children aged 6–12 years. The review included studies with cross-sectional or longitudinal cohorts, compared to a control group of children born at term or to standardized scales. The quality of evidence of the selected studies was verified with the Mixed Methods Appraisal Tool (MMAT). The initial search identified 1,040 articles. Forty articles met the inclusion criteria and were finally included in this review. These studies involved 5,396 preterm children from 37 different cohorts. Despite the diversity found among the results, in general, total IQ scores were within the normative mean for premature children; however, compared to their full-term peers, these scores were lower. The most studied variables in relation to IQ are perinatal (e.g., gestational age and birth weight) and family (e.g., socioeconomic level and education level of the mother). Recent studies corroborate that premature birth affects cognitive development in school age, and identify associated perinatal and family variables.

**Systematic review registration:**

https://www.crd.york.ac.uk/PROSPERO/display_record.php?RecordID=337371; identifier: CRD42022337371.

## Introduction

Premature birth (before 37 weeks of gestation) is associated with certain risks to child development. Around 15 million children per year are born before the pregnancy reaches term, which would mean more than one premature birth for every ten deliveries worldwide. The rate ranges between 5% and 18%, depending on the country, with a higher risk of premature birth observed in low-income countries, especially among the poorest families within the same country (World Health Organization [WHO], 2018). Medical and scientific-technical advances of Neonatal Intensive Care Units have improved the survival rate of premature children considerably in the last decades. Despite this perinatal progress, the short- and long-term comorbidity rates have not decreased so much. Prematurity is often associated with poor motor, cognitive and linguistic development of the child, as well as behavioral problems that affect, among other areas, the child's performance at school (Van Noort-van Der Spek et al., 2012; Moreira et al., 2014; Ong et al., 2015; Allotey et al., 2018). The rate of extremely preterm (EPT) children who show impairment rates in one or more of these neurodevelopmental domains has been reported to be above 70% (Hutchinson et al., 2013). Research on the improvement of their quality of life and the decrease of future health problems must be a priority.

The most adverse outcomes include cognitive problems, which often go unnoticed in early childhood, but emerge at school age in the face of environmental demands, even for children who are not severely disabled (Johnson, 2007). This systematic review is focused on school age outcomes rather than earlier outcomes, since studies at school age are scarce. Most studies have analyzed cognitive outcomes in infancy, when the neonatal medical follow-up programs are still active (Arpi et al., 2019). There is a wide range of cognitive difficulties shown by premature children in the school environment, and they are characterized by their high prevalence and low severity (Johnson, 2007). Cognitive performance is an important element for school children, as it can determine their personal and social adjustment throughout their entire childhood. Successful adaptation to the school context, both in the social and academic scope, may generate positive feelings of self-competence, self-efficacy and, ultimately, personal wellbeing in childhood and adolescence (Verdugo and Sánchez-Sandoval, 2022). The aim of this systematic review was to contribute to the research on cognitive development in premature children. Exploring their cognitive performance during the school stage, as well as the factors related to its functioning, will help to introduce measures that promote a better personal, school and social adjustment at these ages.

For the study of cognitive difficulties, most authors use intelligence and general intellectual functioning, specifically Intelligence Quotient (IQ), as it provides a wider measurement of cognitive functioning and is associated with important achievements in life, such as health, socioeconomic success (Kramer et al., 1995) and academic performance (Martin-Requejo and Santiago-Ramajo, 2021). Previous works have shown that premature birth is associated with cognitive difficulties, and that intelligence is directly proportional to immaturity. Regarding the intelligence scores, significant differences have been reported between premature and full-term children of the same age (Marlow et al., 2005). Premature children show greater rates of developmental delay, worse academic performance and lower mean IQ values than their full-term peers (Ionio et al., 2016; García-Martínez et al., 2018). Recent meta-analyses demonstrate that these differences are detectable in early childhood and persist in adulthood. In the meta-analysis of Arpi et al. (2019), with 13 studies of extremely preterm (EPT) and very preterm (VPT) children aged 3–5 years, in terms of total IQ score, these children scored 0.77 standard deviation (SD) lower than the control full-term children, which poses a decrease of 11.5 points in the total IQ score. In the meta-analysis of Twilhaar et al. (2018b), which included 71 studies with a population aged 5–20 years, the difference between EPT and VPT children and full-term children was −0.89 SD in the total IQ score, which poses a difference of 12.9 IQ points. However, these difficulties in preterm children are not always expressed in results below the normative limits in intelligence evaluation tests (Kerr-Wilson et al., 2012).

Although it is known that the degree of prematurity is a weighty factor for future development, the literature shows that this condition does not pose a specific risk to the child. Individual variation and resilience are characteristics of the preterm population. These findings may be best explained by the confluence of numerous biological and contextual factors. In understanding the factors that affect the cognitive development of preterm children, some of these are specific factors of their condition of prematurity and are related to a shorter gestation or lower weight at birth, as well as to possible neonatal comorbidities, such as bronchopulmonary dysplasia (BPD), periventricular leukomalacia and sepsis. On the other hand, little is known about the role of other factors in the cognitive development that are not specific to the condition of prematurity, such as sociodemographic (e.g., sex and age), family (e.g., parents' education level and income) and social (differences between countries or risk areas) characteristics. An example of the confluence of these factors is shown in the systematic review of 33 studies with preterm children aged 8–10 years (Moreira et al., 2014) with respect to academic, behavioral and motor outcomes. These authors observed that, in addition to biological factors, the analyzed studies found that behavioral disturbances are significantly related to socioeconomic risk factors (socioeconomic status, maternal education and ethnicity), environmental factors (exposure to noise, family conflicts and maternal psychological distress), and motor and developmental components. In this way, changes in the environmental and socioeconomic risk factors could improve the behavior of preterm children.

Therefore, a systematic review was conducted to examine the intelligence of preterm children, in term of IQ scores. Before conducting this review, a search of systematic reviews or recorded protocols was carried out in the International Prospective Register of Systematic Reviews [PROSPERO] (National Institute for Health and Care Research, n. d.), seeking completed or on-going studies on this topic. We found that studies that analyze the cognitive development of preterm children often study other stages of development, such as early childhood, adolescence or adulthood (Raju et al., 2017; Allotey et al., 2018; Brydges et al., 2018). Moreover, some reviews are specifically focused on EPT or VPT, whereas others integrate studies with children born before 37 weeks of gestation. Furthermore, other areas were considered, such as language, motor, social or behavioral development, without specifically considering the cognitive profile and associated variables (Moreira et al., 2014). Other authors have examined the cognitive ability of a specific population of preterm infants with some pathology (Zhou et al., 2021; Pattnaik et al., 2022). Among recent reviews of cognitive outcomes in preterm infants, the meta-analysis by Twilhaar et al. (2018b) stands out, which studied as the main outcome the intelligence of premature infants born in the era of prenatal corticosteroids and surfactants (1990–2008), between the ages of 5 and 20 years. As a limitation, the details of demographic and perinatal risk factors were missing in the analyzed studies, which could be a bias for meta-regression analyses. Differences in the definitions of morbidities (studies did not present the definition they had used) or the measurements could be a possible bias for the incidence rates or the influence of the variables. Additionally, meta-analyses use aggregated data, thereby losing the individual variability of preterm infants. Thus, understanding the profile, the differences and the relationship between preterm child cognitive development and other variables remains a challenge. To our knowledge, few studies have sought the developmental point at which preterm infants begin to match their development to that of full-term infants (López-Hernández et al., 2021). In an attempt to overcome these limitations and to provide clarity to the vital transition of preterm children from early childhood to adolescence, this review was focused on school-age children.

The aim of this work was to summarize available and updated empirical evidence on prematurity as a risk factor for cognitive development in children aged 6 to 12 years. We attempted to identify similarities and differences with the full-term population. As a secondary objective, we aimed to point out possible risk or protective factors (at the biological, psychological or family levels) that may be involved in future evolution.

## Method

The conceptualization and methodology of this review was performed according to the Cochrane system (Higgins and Green, 2012) and PRISMA recommendations (Preferred Reporting Items for Systematic reviews and Meta-Analyses; Page et al., 2021). The review protocol was registered in PROSPERO (CRD42022337371; Sánchez-Sandoval et al., 2022). As indicated in this protocol, the review question was formulated according to the PICOS approach as follows: Are there differences in IQ (Outcome) between preterm (Intervention/Exhibition) and full-term (Comparison) school-aged children (Population)?

### Search strategy and selection procedure

The literature search was carried out in Web of Science, Scopus, PsycInfo and Dialnet databases, which were selected for including journals of impact and relevance in the study field. Following the review question, the Boolean operators (“Preterm” OR “Premature” OR “Premmie birth”) AND (“IQ” OR “Intelligence quotient”) were used in the title and abstract. The search was performed in May 2022, and was limited to journal articles published in the last 10 years in English and Spanish.

**Figure 1:**
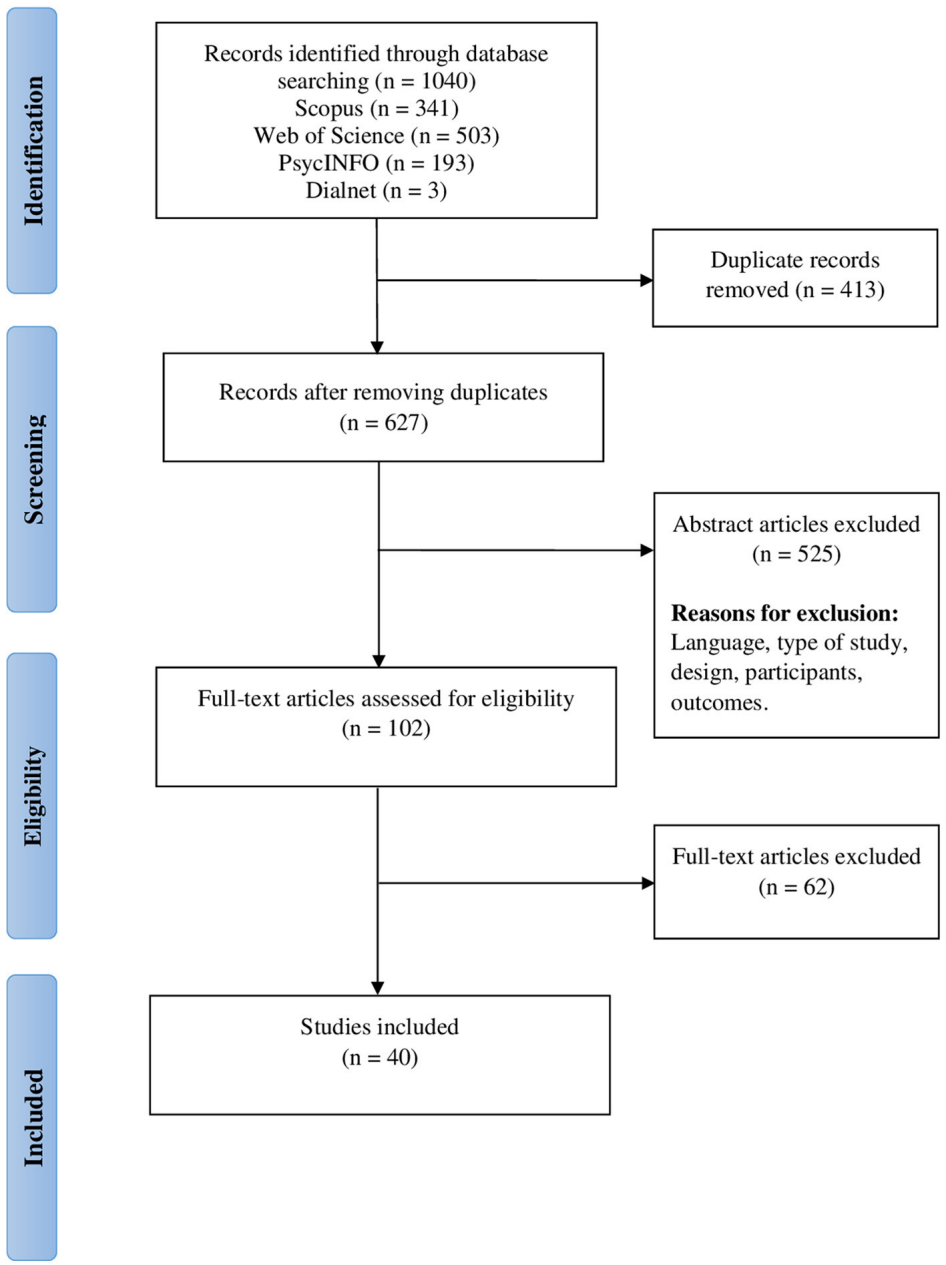
PRISMA flow diagram.

The identified studies were managed with Mendeley. The selection was conducted following the PRISMA indications (Page et al., 2021) (Figure 1). Firstly, duplicate entries were discarded. Subsequently, two independent reviewers read the titles and abstracts and applied the inclusion criteria, and then the full-text articles were read. In case of doubts, a third researcher was involved in the discussion. If the information was insufficient to decide, the authors were contacted.

### Inclusion/exclusion criteria

The inclusion criteria were: (i) experimental, descriptive and correlational studies with cross-sectional or longitudinal cohorts; (ii) samples of children born preterm, under 37 weeks GA, aged 6–12 years at the time of assessment; (iii) outcomes related to cognition measured as IQ using standardized scales; and (iv) comparative results with term-born-children control groups or with normative ranges from standardized scales. The review excluded systematic reviews, meta-analyses and single-case studies, as well as studies with only clinical samples (e.g., with a specific pathology/disability).

### Data extraction and coding

The design of the data extraction table and the data extraction and management were carried out using Microsoft Excel (Pardal-Refoyo and Pardal-Peláez, 2020). To ensure data accuracy, the information was extracted independently by two researchers and then combined after re-checking and reaching consensus. Discrepancies were discussed with a third review author. The files included information regarding: (i) general information, such as author and date; (ii) data about design, measurement instruments and aims; (iii) preterm and full-term samples characteristics; and (iv) main and secondary results with respect to our aims. For the indicator of cognitive performance (IQ), we calculated means, ranges, differences and significance. Moreover, whenever applicable, we included sample distributions as a function of classifications derived from the IQ scores (e.g., average or borderline range).

Once the data were extracted, the results were coded and grouped by similarities to facilitate their synthesis. Regarding the IQ data, we obtained the full test scale mean IQ (FSIQ) score of the premature and control groups, and the mean differences between groups. Other columns were designed to extract the proportions of the sample based on the classification of the normative scale of the instrument used. Furthermore, the rest of the results were organized according to specific cognitive dimensions (means and distributions), correlations of IQ with other individual, psychosocial and family factors, and associations between IQ and other comorbidities or developmental difficulties. The database was created as a function of the results expressed by the reviewed studies (GA, weight at birth, gender, perinatal conditions, brain development, comorbidities, sociodemographic factors and longitudinal associations).

### Risk of bias assessment

The quality of the evidence of the selected studies was verified with the Mixed Methods Appraisal Tool [MMAT; (Hong et al., 2018)]. This tool has recently been used in systematic reviews in psychology (Conejo-Cerón et al., 2021; Gergov et al., 2022), as it is designed for quantitative, qualitative and mixed-methods studies. It includes two screening questions and five items on the representativeness of the sample, the adequacy of the measurements, the value of the data, the design and analysis, and the exposure status. All studies that met the selection criteria exceeded 80% of the MMAT items.

## Results

The initial search identified 1,040 articles (Figure 1), of which 413 were excluded, as they were duplicates. After title and abstract screening, 525 studies were excluded. Common reasons for exclusion were study design, failing to meet subject inclusion criteria, and presenting no analysis of the main outcome (IQ). At this stage, 102 articles were selected for full-text review, and 62 of them were excluded for not meeting the inclusion criteria. Therefore, this systematic review included 40 studies that met all the abovementioned inclusion criteria.

### Sample characteristics

These 40 studies involved 5,396 preterm children from 37 different cohorts. The studies included in this review came from 19 different countries. Concerning the region under study, most of these works were focused on Europe (60%). The Netherlands and Finland were the most frequent countries (six and five studies), followed by Australia (four studies) (Table 1).

**Table 1:** Characteristics of the studies included in the systematic review.

	**Sample characteristics**					**Methodology**		
		**PT group gestation (wk) and birthweight (g)**						
**Reference**	**Country; birth year**	**Inclusion criteria**	**Final sample, mean (SD)**	**Sample size**	**Age at follow-up (y)**	**Recruitment source and study design**	**Term-born group**	**Quality of evidence (MMAT)**
Arhan et al. (2017)	Turkey 1999–2000	29–34 1,500–2,500	29.6 (1.1) 1634 (345)	PT: 22 FT: 24	9	Single-center Case-control Cross-sectional	Matched from the classmates	5/5
Bogičević et al. (2019)	Netherlands 2010-2011	32–36 NR	34.7 (1.3) 2529 (490)	PT: 88 FT: 83	6	Multi-center Case-control Longitudinal	Recruited from the same hospital	5/5
Carmo et al. (2022)	Brazil 2003–2012	≤ 36 NA	30.0 (3.5) 1354.0 (623.5)	PT: 83	6-14	Single-center Normal values Cross-sectional	NA	5/5
Cheong et al. (2017)	Austalia 1991–1992; 1997; 2005	< 28 < 1,000	25.8 (1.1); 25.6 (1.2); 25.8 (1.2) 887 (175); 820 (173); 867 (193)	PT: 468 FT: 571	8	Multi-center Case-control Longitudinal	Recruited at birth from the same hospitals, matched for expected date of birth, sex, mother health insurance status and language.	5/5
Córcoles-Parada et al. (2019)	Spain 1995–2004	< 32 < 1,500	NR NR	PT: 29 FT:14	8-16	Single-center Case-control Longitudinal	Recruited from schools in the same demographic area, matched age and gender	5/5
Cserjési et al. (2012)	Netherlands 2002–2003	32–35 NR	33.9 (1.1) 2239 (489)	PT: 248 FT: 130	7	Single center Case-control Longitudinal	Born in the same center in the same age range	5/5
Dai et al. (2020)	New Zealand 2005–2008	< 30 < 1,500	26 919 (206)	PT: 76	7	Single-center Normal values Cross-sectional	NA	5/5
Domellöf et al. (2020)	Sweden 2000–2005	< 35 NR	31.1 (3.5) 1637 (690)	PT: 51 FT: 57	4-8	Single-center Case-control Cross-sectional	Recruited from the same hospital, matched sex and nearest birth date	5/5
Dubner et al. (2019)	USA 2012–2015	< 32 < 2,500	NR NR	PT: 35 FT: 43	6	Multi-center Case-control Cross-sectional	NR	5/5
Fan et al. (2013)	Brazil 1999–2000	< 37 < 2,500	33.6 (2.0) 1890 (4.9)	PT: 97	6-7	Single-center Normal values Cross-sectional	NA	4/5
Gould et al. (2021)	Australia 2001–2005	< 33 NR	29.3 (2.4) NR	PT: 554	7	Multi-center Normal values Longitudinal	NA	5/5
Grunewaldt et al. (2014)	Norway 1999–2001	< 26 < 1,000	26.3 (1.9) 797 (145)	PT: 23 FT: 33	10	Single-center Case-control Cross-sectional	Age-matched healthy children recruited from four schools in the same area	5/5
Heeren et al. (2017)	USA 2002–2004	< 27 NR	NR NR	PT: 873	10	Multi-center Normal values Cross-sectional	NA	5/5
Hutchinson et al. (2013)	Australia 1997	< 28 < 1,000	26.5 (2.0) 833 (1164)	PT: 189 FT: 173	8	Single-center Case-control Longitudinal	Recruited in the same time period, matched for date of birth, gender, mother's country and health insurance status	5/5
Jin et al. (2020)	South Korea 2006–2011	32–36 NR	34.6 (7.5) 2229.2 (472.8)	PT:37	7-10	Single-center Normal values Cross-sectional	NA	5/5
Joseph et al. (2016)	USA 2002-2004	< 28 NR	NR NR	PT: 873	10	Multi-center Normal values Cross-sectional	NA	5/5
Kaul et al. (2021a)	Sweden 2004–2007	< 27 NR	25.0 (1.0) 782 (168)	PT: 359 FT: 367	6.5	Multi-center Case-control Cross-sectional	Matched recruited from registry	5/5
Kaul et al. (2021b)	Sweden 2004–2007	< 32 NR	28.6 (2.4) 1202 (346)	PT: 91 FT:67	6.5	Single-center Case-control Longitudinal	Results from another national study	4/5
Kim et al. (2021)	Korea 2008–2009	< 30 < 1,000	27.5 (2.2) 885 (238)	PT: 71 FT: 24	7-8	Single-center Case-control Cross-sectional	Recruited via an in-hospital announcement	5/5
Koç et al. (2015)	Turkey 2001	< 32 < 1,500	31.0 (1.7) 1,307 (182)	PT: 90	5-7	Single-center Normal values Longitudinal	NA	5/5
Lind et al. (2020)	Finland 2001–2006	< 32 < 1,500	28.9 (2.7) 1,116 (311)	PT: 167 FT: 149	5;11	Single center Case-control Longitudinal	Recruited from the same study (PIPARI)	5/5
Nagy et al. (2019)	Budapest (Hungary) NR	< 35 < 1,000	27 (2.1); 30 (2.04) 825 (109.6); 1260 (156.1)	PT: 54 FT: 27	9-10	Single-center Case-control Cross-sectional	Recruited from schools and internet.	5/5
Nobre et al. (2020)	Brazil 2012	< 37 ≤ 1,500	31 (2) 1190 (279)	PT: 50	6-7	Single-center Normal values Cross-sectional	NA	5/5
Nyman et al. (2017)	Finland 2001–2006	< 32 ≤ 1,500	28.8 (2.7) 1080 (291.8)	PT: 128	11	Single-center Normal values Longitudinal	NA	5/5
Odd et al. (2012)	Bristol (UK) 1991–1992	< 37 < 2,500	35 (1.2) 2495 (489)	PT:741 FT:13102	8-11	Multi-center Case-control Longitudinal	Data on the control group in another study.	5/5
Qasemzadeh et al. (2013)	Iran NR	< 37 NR	33.74 (1.45) 2226.81 (106.06)	PT: 147 FT: 156	10	Single-center Case-control Cross-sectional	Recruited from the same hospital	5/5
Roberts et al. (2013)	Australia NR	< 32 < 1,500	28.41 (2.41) 1143.12 (313.74)	PT: 258	5;8	Single-center Normal values Cross-sectional	NA	5/5
Roze et al. (2021)	Netherlands 1996–2002 2002–2003	≤ 32 NR	29.4 (NR) 1138 (NR)	PT: 60 FT: 120	6-12y	Multi-center Case-control Cross-sectional	Recruited from 13 preventive child health centers in the Netherlands.	4/5
Squarza et al. (2017)	Milan (Italy) 2002–2007	< 32 < 1,000	27.7 (2.3) 769.7 (165.5)	PT: 99	1;7	Single-center Normal values Longitudinal	NA	5/5
Sripada et al. (2018)	Norway 2003–2007	< 37 ≤ 1,500	29 (2) 1039 (313)	PT: 41 FT: 128	4y-12y	Multi-center Case-control Longitudinal	Recruited from the national Norwegian Mother and Child Cohort Study (MoBa)	5/5
Tanis et al. (2012)	Netherlands 2000–2001	< 32 < 10th percentile	29.7 (NR) 888 (NR)	PT:28 FT:28	8-9	Single-center Case-control Cross-sectional	Recruited from the same hospital	5/5
Teo et al. (2018)	Singapur 1994–1995 2004–2005	< 30 < 1,250	29.4 (2.7); 28.1 (2.5) 1020 (NR); 976 (NR)	PT:145	2;5;8	Single-center Normal values Longitudinal	NA	5/5
Tommiska et al. (2020)	Finland 1996–1997	< 27 < 1,000	27.3 (NR) 802 (NR)	PT: 122 FT: 30	11	Multi-center Case-control Longitudinal	Recruited from a local elementary school, from children participating in the standardization of the neurodevelopment test and children of Hospital personnel.	4/5
Turpin et al. (2019)	Switzerland 1998	≤ 33 NR	30.53 (2.11) 1452.88 (382.85)	PT: 33 FT: 21	11	Single-center Case-control Longitudinal	Recruited from the same hospital	5/5
Uusitalo et al. (2020)	Finland 2001–2006	< 32 ≤ 1,500	29.1 (2.7) 1134.4 (315.3)	PT: 170	11	Single-center Normal values Longitudinal	NA	5/5
Uusitalo et al. (2021)	Finland 2001–2006	< 32 ≤ 1,500	29.01 (2.7) 1119.2 (314.4)	PT: 174	2;11	Single-center Normal values Longitudinal	NA	5/5
van Houdt et al. (2019)	Netherlands NR	< 30 < 1,000	28.13 (1.4) 1080.05 (259.5)	PT: 113 FT: 38	7-12	Multi-center Case-control Cross-sectional	Recruited from friends or family of PT participants, schools and through posters at sports clubs.	5/5
van Veen et al. (2020)	Netherlands 2008–2010	< 30 < 1,000	NA NA	PT: 120	8	Single-center Normal values Cross-sectional	NA	5/5
Wei et al. (2018)	Finland 200–2005	23–33 < 1,000	27.5 (NR) 799.3 (NR)	PT: 84 FT: 86	11	NR Case-control Cross-sectional	Recruited from friends of the cases and from an elementary school close to the examination center	5/5
Young et al. (2019)	Canada 2008–2010	< 32 NA	28.13(1,4) NA	PT:39 FT:34	6	Single-center Case-control Cross-sectional	NR	4/5

PT, Preterm infants (GA < 37 weeks); FT, Full-term (GA > 37 weeks); NR, not reported; NA, not applicable.

The largest samples correspond to Fan et al. (2013), with 873 participants, with both studies being involved in The Extremely Low Gestational Age Newborn (ELGAN) study. The study with the smallest sample of preterm children had 22 participants (Arhan et al., 2017). The mean age of preterm participants in the analyzed studies was between 6.2 (Dubner et al., 2019) and 11.47 (Turpin et al., 2019) years. The female participants represented between 30.4% (Young et al., 2019) and 65.0% (Grunewaldt et al., 2014) in the preterm groups. A set of 25 studies (62.5%) used a cross-sectional research design, and 15 studies (37.5%) used a longitudinal design.

Regarding the characteristics of the preterm sample, the GA range was from 23 to 36 weeks. Based on gestational age (GA), the sub-categories of preterm birth are extremely preterm infants (EPT; GA < 28 weeks), very preterm infants (VPT; 28–32 weeks' GA), and moderate-to-late preterm infants (MLPT; 32–37 weeks' GA). According to this GA classification, a large number of articles (47.5%) included only VPT. To a lesser extent, the rest of the articles were focused on EPT (8/40 studies) and MLPT (4/40 studies). The remaining 22.5% referred to premature infants in general (GA > 37 weeks).

With regard to birth weight, we found information in 27 of the 40 studies. Birth weight ranged between 400 and 3,850 grams, and it was possible to classify them as: extremely low birth weight (ELBW; < 1,000 g), very low birth weight (VLBW;1,000–1,500 g) and low birth weight (LBW; 1,500–2,500 g). Thus, 25% included ELBW preterm, 27.50% VLBW preterm, and 12.5% LBW preterm (Nagy et al., 2019) considered both ELBWs and VLBWs, independently.

Most studies (23/40) had a control group, all of which consisted of full-term children. Most of the studies consider that full-term babies are those born at ≥37 weeks GA with ≥2,500 g birth weight. The total full-term sample included 15,424 participants. The largest number of full-term participants was found in Odd et al. (2012), representing 83.97% of our sample, while the smallest sample was found in Córcoles-Parada et al. (2019), with 14 participants. Children in the comparison group were also assessed during school age, with a mean age between 6.2 (Dubner et al., 2019) and 11.25 years (Turpin et al., 2019). Girls accounted for 30–61.90% of the participants.

### Cognitive outcomes

Table 2 shows the measures that were used to evaluate intelligence. The most commonly used instrument (in 31 studies) was the Wechsler Intelligence Scale for Children, in WISC-IV and WISC-III editions (Wechsler, 1991, 2003). To a lesser extent, other measures of intelligence used in the studies included in this review were Weschler Abbreviated Scale of Intelligence, WASI (Wechsler, 1999), Wechsler Non-Verbal test. WNV (Wechsler and Naglieri, 2008), School-Age Differential Ability Scales II, DAS-II (Elliott, 2007), Verbal and Nonverbal Reasoning scales, Raven's Progressive Matrices (Raven et al., 1998) and NEPSY-II (Korkman et al., 2007).

**Table 2:** Aims and results of the included studies.

	**Aims**	**IQ Measure**	**Results**
Arhan et al. (2017)	To test the hypothesis that regional brain volumes may be associated with long-term cognitive impairments.	WISC-R	Although global intellectual performance was within normal limits in PT children, it was significantly decreased compared with FT children
Bogičević et al. (2019)	To compare MLPT children with FT children in cognitive and behavioral functioning, and to assess whether skills in toddlerhood predict cognitive and behavioral functioning at age 6, similarly for MLPT and FT children.	WPPSI-III	Poorer performance in MLPT children compared to FT children, specifically in processing speed IQ, and mother-rated attention problems
Carmo et al. (2022)	To know the national profile of intellectual disability and school-related difficulties among PT children, and to identify sociodemographic and premature factors related to these outcomes.	WISC-IV	Higher-than-expected incidence of insufficient academic performance in PT children. No association between lower family income, lower maternal schooling and poor performance in WISC or in the psychoeducational evaluations
Cheong et al. (2017)	To compare neurodevelopmental outcomes in an EP cohort with earlier cohorts recruited in the post-surfactant era.	WISC-III; WISC-IV; DAS-II	IQ and academic achievement scores were much higher in controls than in EP children in all cohorts and similar between the EP cohorts from different eras. The gap between the EP children and controls widened over time
Córcoles-Parada et al. (2019)	(…) To determine neurocognitive the outcome of high-order cognitive functions in a subgroup of neurologically healthy children from the VPT-VLBW cohort.	WISC-IV	VPT-VLBW children's mean Full Scale IQ was below control levels, with the perceptual reasoning index being especially affected.
Cserjési et al. (2012)	To compare MLPT children with FT peers on neuropsychological and motor outcomes, paying particular attention to gender differences.	WISC-III	The MLPT group performed more poorly than the FT group on every measure. Using raw scores, there were no gender differences
Dai et al. (2020)	To examine the associations between intelligence, executive function and academic achievement in VPT children.	WISC-IV	The cohort had lower IQ than the normal values, with a 2-fold increased risk of IQ scores −1 SD below the mean
Domellöf et al. (2020)	To explore cognitive and behavioral outcomes relative to GA.	WISC-IV	A main group effect was found for FSIQ, VCI, PRI, and WM, but not for PS, characterized by a lower general cognitive score for PT compared with FT
Dubner et al. (2019)	To describe the corpus callosum in the 3 groups using dMRI. To describe long-term neurocognitive outcomes in the 3 groups.	WASI-II	General linear model revealed a significant main effect of the three groups in IQ. Planned comparisons showed significantly lower IQ in PT+ and PT- compared to FT
Fan et al. (2013)	To assess the cognitive and behavioral development of PT and LBW newborns from disadvantaged social and economic environments.	WISC-III	Borderline results (70 to 80) were observed in 9.3% of children for the Full Scale IQ. A significant association was observed between maternal education/family income and WISC scores.
Gould et al. (2021)	To compare standardized scores for cognitive and motor development according to both chronological and corrected age.	WASI	FSIQ scores were slightly lower when uncorrected compared with scores that were corrected for PT birth
Grunewaldt et al. (2014)	To examine the functional outcome and brain pathology in a cohort of ELBW children without cerebral palsy compared with healthy term-born controls.	WISC-III	ELBW children did not score significantly lower than controls on Full Scale IQ or any of the IQ indices, except for working memory
Heeren et al. (2017)	(…)To compare cognitive profiles based on IQ and EF with a standard classification.	DAS-II	Three-quarters of EP children had normal profiles, whereas 17% had moderately impaired profiles and 8% had severely impaired profiles
Hutchinson et al. (2013)	To investigate cognitive, academic, and behavioral outcomes in EPT/ELBW children.	WISC-IV	The mean FSIQ for the EPT/ELBW group was significantly below that of the T/NBW group. Controlling for sociodemographic variables marginally reduced the mean group difference
Jin et al. (2020)	To evaluate neurodevelopmental outcomes, including cognitive function, executive function, and emotional and behavioral development.	K-WISC-IV	Although statistically insignificant, the mean FSIQ score was lower for MLPT children than for LPT children. We found that early school-aged children showed a lower mean FSIQ compared to the normal population mean
Joseph et al. (2016)	To assess the rate of neurocognitive impairment. To examine the effect of weeks of gestation at birth on the risk of neurocognitive and academic outcomes.	DAS-II	Distributions of test scores were consistently and markedly shifted below normative expectations. Poorer scores were associated with lower GA at birth
Kaul et al. (2021a)	To investigate the cognitive profiles of PT children in detail, investigating mean group differences in Full- Scale IQ and indices.	WISC-IV	Group differences in Full-Scale IQ, indices and subtests were all statistically significant, with medium to large effect sizes
Kaul et al. (2021b)	To compare neurodevelopmental results at 2.5 and 6.5 years in VPT children, and factors related to cognitive impairments.	WISC-IV and WPPSI-III	The differences in mean increased between 2.5 and 6.5 years. Several strong correlations were found between GA, severe brain injury, severe retinopathy of prematurity, treated patent ductus arteriosus, bronchopulmonary dysplasia, and WISC scores
Kim et al. (2021)	To evaluate the cognitive and behavioral outcomes and the risk factors for poor cognitive outcomes.	WISC-IV	The mean FSIQ was significantly lower in the EP group than in the term control group
Koç et al. (2015)	To assess growth, neurodevelopmental and school performance of VLBW and ELBW PT infants.	WISC-R	BW and GA were not correlated with WISC-R scores. Among environmental factors, the paternal education level and occupation correlated significantly with the WISC-R IQ scores
Lind et al. (2020)	To assess the predictive value of intellectual functioning and neuropsychological profile in VPT children at 5 years and to report the neuropsychological profile and risk factors for weaker neuropsychological functions.	WPPSI-R and NEPSY II	Poorer intellectual functioning and weaker neuropsychological functions were related to a need for educational support services. Average neuropsychological performance was consistently poorer in VPT children than the normative mean, yet mostly within the average range. Risk factors for poorer neuropsychological functions were major brain pathology at term-equivalent age, lower paternal education and probably male sex.
Nagy et al. (2019)	To evaluate VLBW/ELBW PT children in basic cognitive abilities and executive function as compared to FT children.	WISC-IV	The mean scores of each measure of the WISC-IV fell within the normal range in all three groups. The ELBW children scored significantly lower in the Full-Scale IQ, Processing Speed and Perceptual Reasoning
Nobre et al. (2020)	To examine the neonatal clinical status, temperament, and attention variables, controlling for socioeconomic variables, to predict cognitive outcomes.	WISC-III	Results show predominantly average classifications in Full IQ, verbal IQ, and performance IQ sections. Predictive model for the Full IQ included as main predictor variables verbal IQ and maternal schooling
Nyman et al. (2017)	To describe the cognitive profile by assessing specific cognitive domains and evaluating the underlying sociodemographic and neonatal risk factors.	WISC-IV	General cognitive performance of the VPT children was within the average range, but significantly lower than the mean test norms. The IQ distribution of the PT population shifted to the left. Low paternal education, male gender, and birth weight z-score were significant risk factors
Odd et al. (2012)	To investigate whether MLPT infants have poorer cognitive, memory, attention, or school outcomes in childhood than those born at term.	WISC-III	Preterm infants had slightly lower verbal, performance, and summary IQ scores than term infants. This association was attenuated after correction for socioeconomic factors
Qasemzadeh et al. (2013)	To survey the relationship between preterm birth and IQ.	Raven test	No significant relationship between age and gestational age and IQ showed a significant direct correlation between weight and head circumference at birth
Roberts et al. (2013)	To examine the effect of age correction on IQ scores and to explore the clinical implications of age correction (…).	WPPSI-III; WISC-IV	Corrected scores were significantly higher than not-corrected scores. No significant correlation was found between IQ score differences and birthweight for either WPPSI-III or WISC-IV scores. There was a weak significant negative correlation between GA and WPPSI-III scores
Roze et al. (2021)	To investigate the co-occurrence of cognitive impairments, comparing these with FT children. To determine whether certain cognitive impairments co-occur more frequently with other cognitive impairments and relate to educational achievement.	WISC-III	PT children performed significantly poorer on all tests except for the visuomotor integration test. The distributions were fairly similar to those in the control group, although the means were lower. The number of PT children with abnormal scores in multiple cognitive domains was higher than in the control group. All PT-born children with an abnormal total IQ had additional cognitive impairments in other domains
Squarza et al. (2017)	To investigate the association between neurodevelopmental quotients at 1 year of corrected age and cognitive functioning at 7 years.	WISC-III (7y); Griffiths Mental Development Scales Revised (1y)	IQs were in the average range at both ages. General Quotient < 1 SD at 1 year of corrected age increases the odds of low IQ scores at 7 years, controlling for biological, neonatal, and family factors
Sripada et al. (2018)	To assess executive function at early school age and examine possible interactions with brain development over time.	WISC-IV; WPPSI-III; WASI	The preterm group showed significantly lower surface area in the parieto-occipital cortex and added effect of IQ on the surface area in PT compared to FT
Tanis et al. (2012)	To measure the outcome of VPT children and SGA in motor, cognitive, and behavioral domains, and compare it with those of school-aged AGA controls.	WISC-III	Total intelligence was significantly lower in the SGA group, but only after we categorized outcomes in this domain
Teo et al. (2018)	To compare the rates of survival, neonatal morbidity, mortality and neurodevelopmental impairment in 2 cohorts.	WISC-III or WISC-IV	Overall neurodevelopmental outcomes over a decade did not worsen despite a lower mean GA. Long-term improvement in IQ scores and a reduction in visual impairment rates were seen. The assessment of neurodevelopmental impairment at 2 years of age may serve as a good cutoff to predict 5- and 8-year outcomes
Tommiska et al. (2020)	To assess cognitive and neuromotor outcomes, attention-deficit hyperactivity (ADHD) features, and school progression in survivors of the population-based cohort of ELBW children, comparing these with FT children.	WISC-III; NEPSY-II	The mean FSIQ was significantly lower than that of the control children. The ELBW children had FSIQ within the normal range, but with a significant difference compared to the control children
Turpin et al. (2019)	To examine the impact of the infant's perinatal risk factors and the mother's post-traumatic stress disorders symptoms due to premature birth on the child's intellectual abilities at 11y.	WISC-IV	Group differences with small effect sizes for age of assessment, IQ and Verbal Comprehension scores. Perinatal factors partly explain preterm-born children's difficulties at birth. Maternal emotional distress appears to be a good predictor of intelligence
Uusitalo et al. (2020)	To evaluate the rate of developmental coordination disorder (DCD) and to study the correlation between motor and cognitive development, and the effect on quality of life.	WISC-IV	VPT children with DCD had lower Full-Scale IQ than VPT children without motor impairment, reporting lower quality of life than VPT children without DCD
Uusitalo et al. (2021)	To study the association between neurological structure at 2 years, neurocognition at 2 years (corrected age) and neurocognition at 11 years in VPT children.	WISC-IV	The neurological structure at 2 years was associated with cognitive development at 11 years. Higher scores at 2 years were associated with better IQ, VCI, PRI and PS at 11 years
van Houdt et al. (2019)	To examine cognitive, behavioral, and academic outcomes in VPT and/or ELBW children with highly educated parents, comparing the results of FT children with those of highly educated parents.	WISC-III	IQ and behavioral functioning were significantly poorer in VPT, but academic functioning was not. Children with only one highly educated parent performed poorer than children with two highly educated parents.
van Veen et al. (2020)	To find possible discrepancies between verbal IQ and performance IQ, and associations with early cognitive outcomes and sociodemographic and neonatal factors	WISC-III	Significant differences were found between verbal IQ and performance IQ. GA, SGA status and cognitive outcomes at 2 and 5 years were important predictors for both at 8 years
Wei et al. (2018)	To test whether there is an association of head circumference or cognitive performance with retinal microcirculatory properties in ELBW preterm children	WNV	ELBW children had a smaller head circumference and narrower retinal venular and arteriolar diameters. IQ was lower and positively correlated with central retinal arteriolar equivalent and arteriole-venule ratio, even controlling for risk factors
Young et al. (2019)	To identify differences in fractional anisotropy within white matter tracts between PT and FT, (..) and associations with developmental outcomes	WASI	White matter differs between VPT and FT children on a microstructural level. In VPT children, intellectual ability, visuomotor skills and early white matter injury were associated with diffusion imaging measures

GA, gestational age; PT, Preterm infants (GA < 37 weeks); EPT: Extremely preterm infants (GA < 28 weeks); VPT, very preterm (EG = 28–32 weeks); MLPT, moderate-to-late preterm (GA = 32–37 weeks); LPT: late preterm (GA = 34–36 weeks); FT, Full-term (GA >37 weeks); BW, Birth weight; ELBW, Extremely low birthweight (< 1,000 g), VLBW, very low birthweight (1,000–1,500 g); LBW, low birthweight (< 2,500 g); T/NBW, term/normal birth weight (>2,500 g); SGA, small-for-gestational-age; AGA, appropriate-for-gestational-age; SD, standar desviation; IQ, Intelligence Quotient; FSIQ, Full-scale intelligence quotient; VCI, verbal comprehension index; PRI, perceptual reasoning index; WM, working memory; PS, processing speed.

A total of 37 articles provide group mean scores in the Full Scale Intelligence Quotient (FSIQ). Scores were obtained between 83.9 (14.6 SD) and 111.1 (10.3 SD) for premature children. The studies with a control group (*n* = 23) reported a FSIQ mean between 100.0 (17.0 SD) and 117.1 (16.4 SD) for full-term children. The three remaining articles did not report mean scores, showing only the distributions between different levels of the test scores (Koç et al., 2015; Joseph et al., 2016; Heeren et al., 2017).

Most of the selected articles showed that school-aged premature children obtained worse total IQ scores than full-term children. Their mean scores were lower when compared with both a control group and the normative values (Table 3). Significant differences have been identified in studies with EPT samples (Hutchinson et al., 2013; Cheong et al., 2017; Wei et al., 2018; Domellöf et al., 2020; Tommiska et al., 2020; Kaul et al., 2021a; Kim et al., 2021), VPT samples (Arhan et al., 2017; Córcoles-Parada et al., 2019; Dubner et al., 2019; Turpin et al., 2019; van Houdt et al., 2019; Young et al., 2019; Dai et al., 2020; Domellöf et al., 2020; Lind et al., 2020; Roze et al., 2021) and MLPT samples (Cserjési et al., 2012; Odd et al., 2012; Bogičević et al., 2019; Domellöf et al., 2020). However, other studies have reported that, although there certain differences between the scores of premature children and those of full-term children, these differences were not statistically significant (Fan et al., 2013; Grunewaldt et al., 2014).

**Table 3:** Descriptive statistics and comparisons between preterm groups and full-term groups, in included studies with a control group.

		**Preterm**	**Full-term**		
		**Mean IQ (SD or range)**	**Mean IQ (SD or range)**	**Mean difference** ***F***, ***t*** **(range)**	***p***
**GA classification**					
All PT	Arhan et al. (2017)	95.68 (10.96)	106.09 (8.06)	−3.22	0.002
	Domellöf et al. (2020)	94.4 (11.1)	102.6 (10.3)	14.39	0.000
	Kim et al. (2021)	91.3 (16.3)	107.1 (12.7)	NR	< 0.001
EPT	Hutchinson et al. (2013)	93.1 (16.1)	105.6 (12.4)	212.5 (−15.5 to −9.5)	< 0.001
	Domellöf et al. (2020)	88.2 (9.9)	102.6 (10.3)	7.58	0.000
	Kaul et al. (2021a)	83.9 (14.6)	100.3 (11.7)	14.8 (12.8 to 16.8)	< 0.001
	Cheong et al. (2017)	94.9 (16.5) 93.8 (14.7) 94.7 (15.7)	104.7 (14.1) 105.6 (12.4) 107.2 (10.9)	−9.6 −11.8 −12.7	NR
	Grunewaldt et al. (2014)	98 (90 to 106)	105 (98-112)	NR	0.208
VPT	Domellöf et al. (2020)	95.6 (9.9)	102.6 (10.3)	NR	NR
	Wei et al. (2018)	93.9 (91.4 to 96.4)	109.2 (106.4–112.1)	NR	< 0.001
	Córcoles-Parada et al. (2019)	101.69 (3.06)	112.57 (3.83)	4.46	0.04
	Turpin et al. (2019)	106.00 (14.74)	114.62 (13.10)	2.185	< 0.05
	Young et al. (2019)	103.04 (11.75)	112.36 (13.43)	−2.53	0.015
	Roze et al. (2021)	92 (55 to 118)	104 (76–132)	NR	NR
MPT	Domellöf et al. (2020)	97.2 (11.6)	102.6 (10.3)	NR	NR
	Cserjési et al. (2012)	101.2 (9.7)	103.9 (10.3)	22.7 (24.8 to 20.6)	0.011
	Odd et al. (2012)	98 (17)	100 (17)	NR	0.087
	Bogičević et al. (2019)	105.1 (13.8) (uncorrected age)	111.4 (12.3)	6.3 (2.3 to 10.3)	< 0.05
**Weight classification**					
ELBW	Nagy et al. (2019)	101.9 (13.8)	112.1 (13.7)	5.33	0.012
	Tommiska et al. (2020)	90 (20)	112 (14)	6.6	< 0.001
VLBW	Nagy et al. (2019)	111.1 (10.3)	112.1 (13.7)	5.33	1.00
	Sripada et al. (2018)	93.5 (9.8)	107.4 (13.8)	NR	< 0.001

GA, gestational age; PT, Preterm infants (GA < 37 weeks); EPT, Extremely preterm infants (GA < 28 weeks); VPT, very preterm (GA = 28–32 weeks); MPT: moderate-to-late preterm (GA = 32–37 weeks); ELBW, Extremely low birthweight (< 1,000 g), VLBW: very low birthweight (1,000–1,500 g); NR, No reference.

In the reviewed studies, the normative range is usually considered to be 85 to 115 points, that is, scores that do not exceed 1SD above or below the normative mean. In general, results showed that premature children obtained scores predominantly in the normative range (Nagy et al., 2019; Nobre et al., 2020). This result has also been found in exclusively EPT samples (Heeren et al., 2017; Tommiska et al., 2020). Kaul et al. (2021a) observed that a third of their EPT sample obtained total IQ scores within the normative range. Similarly, scores have been found mostly in the mean for VPT (Koç et al., 2015; Arhan et al., 2017; Nyman et al., 2017; Squarza et al., 2017; Young et al., 2019; Lind et al., 2020; Uusitalo et al., 2020, 2021). Roze et al. (2021) stated that, although the means were lower, the distribution of the scores of their VPT sample were similar to those of the control group of full-term children. None of the articles with MLPT sample specified the proportion of scores in the normative range. However, two studies (with VPT and EPT samples) showed that the IQ distribution shifted to the left, compared to normative values (Joseph et al., 2016; Nyman et al., 2017).

The studies used different criteria to define the existence of deficit in cognitive development. Following the test criteria, most of them define it through total IQ scores under percentile 10, under 85 points or −1SD. The scores within the range of 70–84 are considered moderate cognitive functioning, which is also called moderate cognitive impairment or borderline (which rather refers to scores between 70 and 79). Scores of < 70 or −2SD are classified as low functioning, which is also called severe cognitive impairment. Considering this classification, as is shown in Table 4, the results showed that between 9% and 39% of the premature sample had moderate or low cognitive functioning (Fan et al., 2013; Koç et al., 2015; Joseph et al., 2016; Heeren et al., 2017; Nyman et al., 2017; Jin et al., 2020; Lind et al., 2020; Nobre et al., 2020; Tommiska et al., 2020; Uusitalo et al., 2020; Kim et al., 2021; Carmo et al., 2022). Some studies specified that the percentage of children with deficit scores in FSIQ was significantly greater in premature children than in full-term children, both compared to the control group (Hutchinson et al., 2013; Kaul et al., 2021a,b; Kim et al., 2021; Roze et al., 2021) and with respect to normative values (Cserjési et al., 2012; Hutchinson et al., 2013; Joseph et al., 2016; Dai et al., 2020). This is in line with the findings of Odd et al. (2012) in a sample of late preterm children.

**Table 4:** Percentages of deficit classification derived from total IQ scores by gestational age.

**GA classification**		**Classification**	**Scores**	**Sample (%)**
All PT	Fan et al. (2013)	Borderline	70–80	9.3%
	Nobre et al. (2020)	Borderline extremely low	70–79 < 69	30%
	Carmo et al. (2022)	Low or borderline	< 80	10.9%
EPT	Tommiska et al. (2020)	Mild Moderate Severe	70–85 55–69 < 55	20% 14% 3%
	Kim et al. (2021)	Risk Borderline	< 85 < 70	36.6% 12.6%
	Joseph et al. (2016)	NR	70–85 < 70	18 to 39% 15 to 34%
	Heeren et al. (2017)	Moderately impaired Severely impaired	70-85 < 70	19% 15%
	Nyman et al. (2017)	Borderline Extremely low	NR	20% 13%
VPT	Nyman et al. (2017)	Borderline extremely low	NR	18% 10%
	Lind et al. (2020)	Neurodevelopmental impairments	< 70	11.37%
	Uusitalo et al. (2020)	Low average Borderline Severe cognitive impairment	80–89 70–79 < 70	21.1% 15.5% 8.1%
	Koç et al. (2015)	Borderline Low	70–89 < 70	32% 14%
MLPT	Jin et al. (2020)	Borderline intelectual functioning Intellectual disability	70–84 < 70	0% 24.3%

In addition to the total scores of the scales (FSIQ), the reviewed studies analyzed a variety of dimensions, domains or cognitive indices. Since the most used scale was WISC, this review is focused on the dimensions of this test: verbal comprehension index (VCI), perceptual reasoning index (PRI), working memory index (WMI) and processing speed index (PSI). Most of the authors found significant mean differences in all indices (Cserjési et al., 2012; Hutchinson et al., 2013; Domellöf et al., 2020; Kaul et al., 2021a; Kim et al., 2021; Roze et al., 2021). On the other hand, one of the studies reported no differences between groups in any of the analyzed indices (Grunewaldt et al., 2014) (Table 5).

**Table 5:** Means and comparisons between preterm groups and full-term groups by WISC dimensions.

		**VCI** ***M*** **(SD)**			**PRI** ***M*** **(SD)**			**WMI** ***M*** **(SD)**			**PSI** ***M*** **(SD)**		
		**PT**	**FT**	**Dif**.	**PT**	**FT**	**Dif**.	**PT**	**FT**	**Dif**.	**PT**	**FT**	**Dif**.
**GA CLASSIFICATION**													
All PT	Carmo et al. (2022)^a^	100.0 (13.9)			99.0 (15.6)			90.0 (13.3)			93.3 (14.4)		
	Domellöf et al. (2020)	96.0 (10.1)	102.8 (10.3)	10.21 (0.002)	101.1 (14.2)	109.5 (11.3)	11.43 (0.001)	87.3 (12.0)	92.6 (11.2)	5.63 (0.019)	95.3 (14.0)	97.4 (12.2)	0.46 (0.501)
	Fan et al. (2013)^a^	98.6 (12.9)			98.8 (16.4)			NR			94.4 (12.8)		
	Kaul et al. (2021b)	99.8 (14.6)	105.6 (11.2)	NR (>0.001)	99.6 (15.8)	103.2 (10.0)	NR (>0.05)	85.3 (13.4)	90.2 (11.6)	NR (>0.001)	92.2 (14.8)	94.2 (11.6)	NR (>0.05)
EPT	Grunewaldt et al. (2014)^a^	109.0 (98.1)			104.0 (92.1)			103.0 (91.1)			112.0 (100.1)		
	Hutchinson et al. (2013)	93.1 (14.3)	103.2 (12.6)	−7.1 (< 0.001)	95.9 (16.8)	108.2 (12.8)	−7.7 (< 0.001)	94.0 (16.3)	102.4 (12.9)	25.5 (< 0.001)	94.7 (15.9)	101.1 (11.9)	24.3 (< 0.001)
	Kaul et al. (2021a)	92.2 (14.4)	104.0 (11.5)	10.0 (< 0.001)	89.7 (14.2)	104.8 (12.7)	13.8 (< 0.001)	78.3 (13.1)	90.7 (11.0)	11.2 (< 0.001)	85.0 (14.4)	96.9 (12.7)	11.5 (>0.001)
	Kim et al. (2021)	94.2 (16.5)	108.5 (11.5)	NR (>0.001)	91.7 (19.7)	108.5 (15.1)	NR (>0.001)	91.2 (16.4)	103.0 (12.6)	NR (>0.001)	86.3 (18.1)	99.0 (15.1)	NR (>0.001)
VPT	Nyman et al. (2017)^a^	90.6 (14.9)			92.9 (16.2)			93.5 (17.0)			93.9 (17.0)		
	Roberts et al. (2013)^a^	90.44 (12.5)			94.11 (14.9)			92.69 (13.9)			93.24 (15.0)		
	Turpin et al. (2019)	110.0 (13.3)	119.8 (12.3)	2.7 (< 0.001)	104.12 (14.9)	108.10 (16.03)	0.92 (>0.005)	96.85 (15.2)	102.14 (9.1)	1.59 (>0.05)	104.45 (16.5)	109.67 (13.4)	1.21 (>0.05)
	Uusitalo et al. (2020)^a^	92.1 (13.4)			94.8 (14.6)			95.4 (15.0)			96.5 (15.5)		
	Uusitalo et al. (2021)^a^	89.8 (14.9)			91.6 (17.3)			92.2 (16.7)			93.6 (17.6)		
MPT	Bogičević et al. (2019)	104.7 (13.5)	110.2 (12.5)	NR (>0.05)	NR			NR			96.0 (14.8)	104.0 (13.3)	NR (< 0.001)
	Jin et al. (2020)^a^	92.70 (10.2)			101.5 (11.1)			92.86 (14.6)			92.43 (13.1)		
**Weight classification**													
ELBW	Nagy et al. (2019)	107.9 (10.9)	112.6 (12.5)	2.4 (0.336)	100.9 (14.1)	109.8 (12.6)	3.46 (0.031)	98.3 (13.0)	105.7 (15.5)	2.97 (0.130)	96.4 (13.5)	107.8(10.5)	6.8 (0.004)
VLBW	Nagy et al. (2019)	114.0 (8.1)	112.6 (12.5)	2.4 (1.00)	105.9 (10.2)	109.8 (12.6)	3.46 (0.750)	106.1 (12.6)	105.7 (15.5)	2.97 (1.00)	107.0 (13.7)	107.8(10.5)	6.8 (1.00)

Dif., Mean difference *F, t (p);* GA, gestational age; PT, Preterm infants; EPT, Extremely preterm infants (GA < 28 weeks); VPT: very preterm (GA = 28–32 weeks); MPT, moderate-to-late preterm (GA = 32–37 weeks); ELBW, Extremely low birthweight (< 1,000 g), VLBW, very low birthweight (1,000–1,500 g); NR, No reference. Only articles that provided information on these dimensions were included.

^a^Does not compare with FT control group.

Specifically, regarding verbal comprehension (VCI), when compared with a full-term group, the studies reported lower scores in preterm children (Hutchinson et al., 2013; Grunewaldt et al., 2014; Turpin et al., 2019; Domellöf et al., 2020; Kaul et al., 2021b). VCI mean ranged between 89.8 (14.9 SD) and 110.03 (13.37 SD) for PT children, whereas, for FT, it ranged between 102.8 (10.3 SD) and 119.81 (12.33 SD). Three papers analyzing the distribution of the preterm group found 14% (Nobre et al., 2020) and 6.2% (Fan et al., 2013) of borderline scores (< 1 SD) and 10% (Nobre et al., 2020) and 5% (Kaul et al., 2021a) of extremely low scores (< 2 SD) in this dimension. Fan et al. (2013) stated that the lowest scores with respect to the standardized scores occurred in this area. However, Kaul et al. (2021a) identified that the strength of their EPT sample was the verbal index.

Regarding perceptual reasoning, some studies indicated that the PRI of preterm children was especially affected (Córcoles-Parada et al., 2019; Kaul et al., 2021a ). Significant mean differences were observed between groups of PT and FT (Cserjési et al., 2012; Hutchinson et al., 2013; Domellöf et al., 2020; Kaul et al., 2021a; Kim et al., 2021; Roze et al., 2021). The range of mean scores in PRI was between 89.7 (14.2 SD) and 104.12 (14.95 SD) for PT and between 103.2 (10.0 SD) and 109.8 (12.6 SD) for FT.

In terms of working memory, the scores found in the comparison with the control group were lower than expected (Odd et al., 2012; Hutchinson et al., 2013; Grunewaldt et al., 2014; Córcoles-Parada et al., 2019; Domellöf et al., 2020; Kaul et al., 2021a), except for one case, where average scores were reported (Nobre et al., 2020), and another case, in which similar scores were obtained between preterm and full-term children (Odd et al., 2012). The mean scores of PT in this index ranged between 78.3 (13.1 SD) and 103 (91.11 SD), whereas the mean scores of FT ranged between 90.2 (11.6 SD) and 105.7 (15.5 SD).

Regarding processing speed, several articles highlighted significant differences with respect to the full-term children (Cserjési et al., 2012; Fan et al., 2013; Hutchinson et al., 2013; Bogičević et al., 2019; Domellöf et al., 2020; Kaul et al., 2021a; Kim et al., 2021; Roze et al., 2021). However, one study found that EPT children with < 2 SD on FSIQ usually showed strength in this area (Kaul et al., 2021a). PSI studies reported a range between 85.0 (14.4 SD) and 112 (100.12 SD).

In regard to the co-occurrence of cognitive deficits in the different domains, Roze et al. (2021) analyzed it in a sample of VPT children, finding that 45% had a result of < 70 in at least one index, and that 15% had a result of < 70 in two or more domains. Moreover, they observed that most of the sample had results of < 85 in multiple domains. In this sense, Kaul et al. (2021a) detected that, among the EPT children with total mean scores, 2% had moderate or severe deficit in only one index, and 40% had moderate or severe deficit in multiple indices. Kaul et al. (2021b) reported that 57.1% and 17.8% of EPT children and 36.5% and 4.8% of VPT children obtained scores below 1 SD and 2 SD, respectively, in two or more indices. Heeren et al. (2017), in a similar study with EPT, found that, among the children with medium and medium-low scores, 1% and 4% of them, respectively, did not show *impairment* in any domain or presented it in only one domain. On the other hand, those children with profiles of moderate or severe global development showed high levels of impairment in all IQ measures. However, none of the authors managed to identify a co-occurrence pattern for these deficits in specific domains.

### IQ in relation to gestational age, weight at birth and gender

Gestational age was a widely studied variable in relation to IQ. Studies found that GA was positively related to IQ scores (Hutchinson et al., 2013; Joseph et al., 2016; Cheong et al., 2017; Heeren et al., 2017; Domellöf et al., 2020; Kaul et al., 2021b; Carmo et al., 2022) and could be an important predictor of cognitive functioning in school age (Domellöf et al., 2020; van Veen et al., 2020). Other authors, although in lower proportion, did not observe significant relationships between GA and IQ (Fan et al., 2013; Qasemzadeh et al., 2013; Roberts et al., 2013; Koç et al., 2015; Nagy et al., 2019; Kim et al., 2021).

Tommiska et al. (2020) observed the distribution in the normative range as a function of GA in ELBW children under 27 weeks (GA). They reported that none of the children born at 22 or 23 weeks (GA) was within the normative range. From week 24 (GA), the number of children with normative development increased with GA. Between weeks 24 and 26 (GA), a third of the ELBW children presented normal cognitive skills. From week 27 (GA), 53% were classified within the normal range. In this line, Heeren et al. (2017) reported that it was more probable for children born at 23–24 weeks (GA) to present severe impairment. Hutchinson et al. (2013) found differences between the results of two subgroups of EPT children of 26–27 weeks (GA) and 27 weeks (GA), with significant differences in the PRI dimension. Jin et al. (2020) also observed these differences between two groups of MLPT children: 32–33 weeks (GA) and 34–36 weeks (GA).

Some studies compared the results of the same sample according to corrected and uncorrected age. IQ scores were significantly higher when age was corrected than when age was not corrected (Roberts et al., 2013; Gould et al., 2021). If age was corrected, 22.1% (*p* < 0.001) of the children who had been classified as “at risk” were no longer in that category (Gould et al., 2021). Bogičević et al. (2019) found that the MLPT children with uncorrected scores obtained worse results of total IQ than the FT children, which was not observed with corrected scores.

Authors also investigated the relationship between weight at birth and cognition. Some of them found a positive relationship, that is, they showed that the higher the weight, the better the outcomes (Qasemzadeh et al., 2013; Cheong et al., 2017), and weight at birth was also considered an important predictor of cognitive development. Tommiska et al. (2020) identified significant differences in the proportion of ELBW children in the normative range (62%) with respect to the control group (100%). Kim et al. (2021) observed that the children who obtained IQ scores < 85 were significantly smaller for their GA or significantly lighter at birth or upon discharge from the NICU. Sripada et al. (2018) also detected that the VLBW participants had lower IQ by approximately 1 SD. More specifically, Hutchinson et al. (2013) identified differences in the results between two subgroups of children with EBLW (< 750 g or 750–999 g). On their part, the differences between the children born small for their GA (SGA) and those with adequate weight for their GA (AGA) were smaller in the study of Tanis et al. (2012). Despite these results, other authors did not find significant differences between the weight at birth and IQ (Fan et al., 2013; Roberts et al., 2013; Koç et al., 2015; Domellöf et al., 2020; Kaul et al., 2021b).

In contrast, Nagy et al. (2019) reported that the SGA children scored higher in the WISC-IV VCI. They found significantly lower scores in the PRI and PSI of ELBW children with respect to the VLBW group. Other works also found a significant association between weight at birth and PSI scores (Hutchinson et al., 2013; Nyman et al., 2017; Carmo et al., 2022).

Regarding gender differences, no significant differences were found between girls and boys by Nagy et al. (2019). However, other authors did find a relationship between sex and IQ (Nyman et al., 2017; Dai et al., 2020), stating that the male gender was associated with lower scores. Specifically, Nyman et al. (2017) obtained this result in cognitive subscales, such as working memory and processing speed.

### Perinatal conditions in relation to IQ

With regard to perinatal factors, we found mixed results concerning IQ. Some results show that there were certain perinatal conditions or characteristics that can be adverse for the cognitive development of the premature infant. Higher Perinatal Risk Inventory (PERI) scores were associated with lower IQ scores (Turpin et al., 2019), significantly affecting VCI. Moreover, variables such as head circunsference at birth, sepsis, necrotizing enterocolitis, longer duration of antibiotic, treated oersistand ductus arteriosus, laser treatment for retinopathy of prematurity (ROP) or severe ROP, height at discharge from the NICU, bronchopulmonary dysplasia, intraventricular hemorrhage, cystic periventricular leukomalacia, postnatal corticosteroids, surgery in the newborn period and perinatal asphyxia (Qasemzadeh et al., 2013; Koç et al., 2015; Cheong et al., 2017; Nagy et al., 2019; Kaul et al., 2021b; Kim et al., 2021) correlated significantly and negatively with IQ scores. Another example would be the study of Nagy et al. (2019), where children with BPD obtained lower scores. Koç et al. (2015) also observed that all the VPT children of their sample with IQ < 85 obtained < 6 points in APGAR at 5 min, and all those with a score of over 6 points showed IQ > 85. On their part, Kim et al. (2021) identified laser treatment for ROP and low discharge weight Z-score as independent risk factors for low FSIQ in the EP cohort. However, the administration of antenatal steroids was associated with significantly better outcomes in the EPT group by Kaul et al. (2021b).

Nevertheless, neonatal clinical variables, which are also called neonatal risk factors, were not predictors of cognitive outcomes (Córcoles-Parada et al., 2019; Nobre et al., 2020; van Veen et al., 2020). Thus, some authors did not find significant differences with respect to APGAR, type of birth, multiple birth, early or late sepsis, or HPIV (Kaul et al., 2021b; Carmo et al., 2022). Other authors did not detect a relationship between intraventricular hemorrhage and retinopathy of prematurity (Nagy et al., 2019) or inflammatory conditions and IQ (Dubner et al., 2019).

Some studies included multiple cohorts in their samples. Cheong et al. (2017) made comparisons between a cohort of EPT born in the post-surfactant era and two previous cohorts born in the 1990's. Regardless of the GA, IQ scores and ratios of < -2SD were similar among the three groups. A small effect was observed when controlling for perinatal variables. Similarly, Teo et al. (2018) found that the IQ scores were significantly higher for the cohort of children born in the mid-2000's, compared to those born in the mid-1990's. A greater proportion of children in mid-2000's had a normal IQ score, although this was not statistically significant and there were no differences in impairment ratios.

### Brain development in relation to IQ

The number of studies that showed MRI data decreased significantly after discarding the articles that were only conducted with clinical samples. However, we found 8 articles on MRI outcomes. Grunewaldt et al. (2014) included cognitive and magnetic resonance results in their study, although they did not examine correlations between both results. The rest of the studies identified significant relationships between MRI findings and IQ. In this sense, one of the most outstanding results was reported by Nyman et al. (2017), who detected that the only significant risk factor for poor general cognition was major brain MRI pathology at term age. In the study of (Dubner et al., 2019), significant positive correlations between mean occipital fractional anisotropy (FA) and IQ scores appeared in the combined sample of full-term and preterm participants. Likewise, Young et al. (2019) indicated a significantly greater association between FA and IQ for VPTs than for those born at term. They found significant associations between numerous white matter areas and IQ, with both DTI and NODDI metrics, for VPT infants. The association between mean diffusivity (MD), axial diffusivity (AD), and radial diffusivity (RD) was significantly higher for full-term infants than for very preterm infants. For VPT children, researchers observed many areas of white matter with significant associations for diffusion tensor imaging (DTI) and NODDI metrics with IQ. Higher IQ scores in this group were significantly associated with higher FA and NDI indices. In contrast, lower IQ scores were associated with lower MD, AD, and RD.

In terms of brain area, some studies have found positive correlations between intelligence test scores and brain volume in certain areas (Arhan et al., 2017; Sripada et al., 2018). Specifically, these studies established positive associations for IQ with total brain volume, with reductions in the cerebellum, hippocampus, and corpus callosum, with greater surface area in the left hemisphere regions of the parieto-occipital and inferior temporal cortex, and with larger volumes of putamen and globus pallidus, respectively. On the other hand, the mentioned studies found negative correlations with cortical thickness in several brain areas, such as frontal pole, medial prefrontal cortex, anterior cingulate cortex, left inferior frontal gyrus, sensory and motor areas, dorsal and posterior insular cortex, posterior superior temporal gyrus, and extrastriate visual cortex (Córcoles-Parada et al., 2019). On their part, the PIPARI study detected that a major brain pathology at term equivalent age was associated with poorer scores (Nyman et al., 2017; Lind et al., 2020).

### IQ and comorbidities

Seventeen studies related intelligence to different comorbidities: disability, brain volume or other brain pathologies, executive function problems, neurodevelopmental or language delay, learning difficulties, academic achievement and behavioral problems. van Veen et al. (2020) found significant associations for neurodevelopmental and language delay at early ages with low WISC scores.

Four authors highlighted the relationship between IQ and the academic scope. A low IQ was related to a below-average performance in reading, writing and mathematics, and greater probability of presenting below-average academic performance (Heeren et al., 2017; Dai et al., 2020). Koç et al. (2015) reported a correlation between academic achievement and IQ, specifically with respect to the classifications they obtained, stating that there were significant differences in the IQ of children who had received special education compared to those who had not. They also observed that the cognitive scores were significantly lower for the children who had started school later and had not attended pre-school education. Roze et al. (2021) found that the children who had repeated a year or received special education presented more domains with scores < 85, although this was detected both in the VPT group and in the control group of FT children. Verbal IQ, performance IQ, visuomotor integration and attention were significantly more frequently affected in these children. Fan et al. (2013) reported a significant association between IQ and schooling.

Uusitalo et al. (2021) analyzed the neurological development in relation to IQ, including children with cerebral palsy (CP) in their sample. They identified that neurotypical development at 11 years of age was associated with high IQ scores, but they also observed that, among the children with CP, 44% obtained scores of 70 or higher. The score under 70 were more common in the children with complex minor neurological disfunction, who obtained lower scores in PRI and PRI and WMI. Koç et al. (2015) specified that a diagnosis of neurodevelopmental delay in the first 3 years of age was significantly correlated with low cognitive scores, as well as delay in speech or speech disorders. Some studies reported that the statistical conclusions remained unaltered after discarding the children who presented disabilities (Hutchinson et al., 2013; Nyman et al., 2017; Kim et al., 2021).

On the other hand, some works analyzed the influence of motor impairment. A positive correlation was found between MABC test scores and IQ, WMI, PSI and PRI index by Uusitalo et al. (2020). Preterms (in their case, VPT) with motor impairment had lower full-scale IQ scores and all index scores than preterm infants without impairment. Grunewaldt et al. (2014) found that low scores in WMI and PSI at 10 years of age were related to abnormal motor repertoire in childhood, although they did not observe this in the total IQ index.

In the behavioral area, Domellöf et al. (2020) found a significant association between WISC-IV and CBCL (6–18), relating low scores in working memory to high scores in attention/hyperactivity problems. Fan et al. (2013) also showed a significant association for FSIQ with the social competence domain and total behavior. However, in Jin et al. (2020), CBCL did not report significant associations with neuropsychological results (e.g., FSIQ).

### Sociodemographic factors in relation to IQ

In addition, some studies attempted to demonstrate the existence of a relationship between family factor and cognitive outcomes. Variables such as parental education, socioeconomic status, family income, occupation and post-traumatic stress disorder (PTSD) symptoms were studied in relation to different aspects of children's cognitive development. Some studies state that, when controlling for sociodemographic variables, the differences between groups decreased, although they continued to be significant (Odd et al., 2012; Hutchinson et al., 2013; Kim et al., 2021).

The most frequently considered variable was parents' education. Results mainly showed a positive significant association with level of parents' education for preterm children's cognition scores (Fan et al., 2013; Nagy et al., 2019; van Veen et al., 2020), mainly mother's education, which might be a main predictor of IQ according to Nobre et al. (2020). Lower mother's education was associated with lower IQ (Cheong et al., 2017; Nobre et al., 2020) and higher IQ scores were associated with highly educated mothers (Domellöf et al., 2020). In turn, Odd et al. (2012) found little evidence of the influence of mother's education to modify the relationship between prematurity (in their case, GA) and IQ. In fact, some articles indicate that mother's education reduced the significance of the neonatal variables (Kaul et al., 2021b). Moreover, higher father's education was associated with higher IQ scores (Koç et al., 2015), and lower father's education with lower VCI (Nyman et al., 2017). Cserjési et al. (2012) found slight increases when repeating the analyses controlling for parents' education level, but without statistical significance. Furthermore, if only one of the parents presented a high education level, the IQ of the premature child was significantly lower than if both parents had a high level (van Houdt et al., 2019). Other authors found no significant associations or differences between parents' education level and IQ (Young et al., 2019; Kim et al., 2021).

Studies on socioeconomic status revealed a positive correlation with IQ scores. Children of lower socioeconomic status had a lower mean than those of higher socioeconomic status (Cheong et al., 2017). However, Odd et al. (2012) obtained little evidence of the influence of the mother's socioeconomic status. Specifically, the analysis of family income yielded disparate results, finding an association with IQ in Fan et al. (2013), and between parents' occupation and IQ scores in Koç et al. (2015), but no differences were identified between groups in the recent studies of Carmo et al. (2022) and Kim et al. (2021).

Additionally, one study considered IQ with respect to the emotional aspects of the parents of mothers of premature children, more specifically, the post-traumatic stress syndromes they presented after the birth of their children, which were measured with the Perinatal Post-traumatic Stress Questionnaire (PPQ). The mentioned study found that the scores of the mothers in this questionnaire were negatively and significantly correlated with the IQ scores of school-aged VPT children. Furthermore, they observed that this result was not significant for full-term children, and that the mother's emotional distress could be an even better predictor of intelligence than perinatal factors. Another finding was that the parents' anxiety was significantly higher in children with IQ < 85 (Koç et al., 2015).

### Longitudinal design studies

Lastly, we would like to highlight that, among the longitudinal design studies, five studies used regression analysis to identify predictors of IQ (Squarza et al., 2017; Bogičević et al., 2019; Turpin et al., 2019; van Veen et al., 2020; Kaul et al., 2021b). Children's cognitive abilities during early childhood significantly predicted full-scale IQ variations in school age. These were measured with Bayley at 2 years and WPPSI at 5 years. Neonatal factors such as GA and small for GA were also important predictors of IQ. Other neonatal factors did not improve the explanatory model. Regarding family factors, it was found that mother's education does not independently predict the cognitive functioning of the premature child. However, mother's emotional distress was a better predictor than perinatal factors. Other studies also observed positive significant associations between IQ at 2 or 5 years and IQ during the school period (Nyman et al., 2017; Teo et al., 2018; Bogičević et al., 2019; van Veen et al., 2020; Uusitalo et al., 2021). For instance, Teo et al. (2018) showed that the patients who were categorized as without neurodevelopmental impairment at 2 years of age continued to be unimpaired at 5 and 8 years of age, and only one third of those who were categorized as with neurodevelopmental impairment at 2 years of age continued to be impaired.

## Discussion

The aim of this systematic review was to provide a comprehensive overview of the literature concerning cognitive outcomes during school age in preterm-born children. Despite the difficulty of maintaining cohort studies over time, this review brings together 40 studies conducted in the last 10 years that met the inclusion criteria. These investigations involved 5,396 preterm children. We incorporated studies with two possible designs to compare preterm infants and full-term infants: data from general population and/or data from comparison groups. Taken together, the results confirm an association between preterm birth and intelligence.

Firstly, as a group, the analyzed studies show that preterm children obtained worse IQ results than their full-term peers in school age. These results support previous research. The meta-analysis of (Kerr-Wilson et al., 2012) indicated that preterm birth is associated with a 12-point reduction in IQ score. These lower mean scores in preterm children were also reported by other studies carried out with premature children in early childhood and adolescence (Twilhaar et al., 2018a,b; Arpi et al., 2019) and in adulthood (Eves et al., 2021). Research has shown stable cognitive performance from early childhood to adolescence (Doyle et al., 2015). In addition, there appears to be little cognitive recovery, as other works show only a slight association between age at assessment and cognitive impairment. Preterm children fail to catch up with their term-born peers throughout childhood and adolescence. In this regard, Brydges et al. (2018) inferred that preterm-born children suffer from a deficit in cognition, not a delay. On the other hand, longitudinal studies indicate that the association between infant IQ and preterm birth does not seem to have changed in recent decades despite improvements in neonatal practice (Cheong et al., 2017; Twilhaar et al., 2018b). Scientific and technological advances have substantially increased survival rates after premature birth, but there is still a long way to go to improve the development and quality of life of these children.

Although comparisons of mean IQ scores reveal that the preterm group is at a cognitive disadvantage with regard to their peers, it should be noted that the general cognitive performance of children born preterm was predominantly in the middle range (Koç et al., 2015; Arhan et al., 2017; Heeren et al., 2017; Nyman et al., 2017; Squarza et al., 2017; Nagy et al., 2019; Young et al., 2019; Lind et al., 2020; Nobre et al., 2020; Tommiska et al., 2020; Uusitalo et al., 2020, 2021). The results of these studies show that the IQ scores of the preterm groups did not exceed 1 SD below or above the normative mean, thus ranging from scores of 85 to 115. In any case, their proportion in the medium-low range is higher than expected. In addition, if we check the leftmost end of the normality curve, which represents borderline or low cognitive functioning, the studies agree that the percentage of premature infants in this range is somewhat higher than expected (Cserjési et al., 2012; Hutchinson et al., 2013; Joseph et al., 2016; Dai et al., 2020; Kaul et al., 2021a,b; Kim et al., 2021; Roze et al., 2021). Preterm infants were at increased risk of clinically significant cognitive scores at age 6 to 12, although this is not the norm. We believe this is an important finding. No major deficits have been found, although a somewhat lower level of general intelligence is shown. We conclude that, as these are not major deficits, they may not be detectable in early childhood, and therefore psychoeducational remedial resources are not applied. This slightly lower intellectual performance is more evident in middle childhood, when school demands at the academic level are probably higher. This lower cognitive level may not only have an impact on academic performance, but also on other activities of daily living. In this sense, premature birth would be a risk for the adaptation of these children to different contexts and, finally, for their quality of life. In some countries, such as Spain, Early Intervention is aimed at the first years of life and ends at the age of 6 years at the latest. This means that children whose early need is not detected will not receive this intervention during the first years of life. These lower cognitive skills, which, as shown in this systematic review, preterm children manifest on average during school years, may no longer be associated by teachers and other professionals with being born prematurely, thus they may not receive appropriate intervention. The open design of this review has shown that, although a cognitive deficit becomes evident in school age, it can be predicted in early childhood (Nyman et al., 2017; Teo et al., 2018; Bogičević et al., 2019; van Veen et al., 2020; Uusitalo et al., 2021). This would allow implementing preventive interventions for the improvement of premature children's cognitive development.

Taking these results together, one of the conclusions of this review is that the cognitive weaknesses presented by preterm school-aged children have a high prevalence but low intensity. A lack of maturity at birth seems to be the origin of the adverse neurological outcomes and the differences with respect to full-term peers. There is strong evidence that the brain of the premature infant is highly vulnerable to the occurrence of cerebral white matter injury (Khwaja and Volpe, 2008). Cerebral white matter injury, which is characterized by loss of premyelinating oligodendrocytes, is the most common brain disease in this population and has been associated with the presence of cognitive deficits, including IQ. This is because white matter tracts play an important role in functional connectivity.

Even though intelligence tests are important measures of cognitive functioning, they do not provide data on specific cognitive difficulties. There is a risk that a global IQ score may mask subtle or localized deficits (Matthews et al., 2018; Pascoe et al., 2021). We have attempted to compensate for this bias, and have systematized in this review the results in relation to specific subdomains measured in the intelligence tests. These subdomains have been analyzed less frequently than the overall IQ score. Nevertheless, the analyzed studies mostly coincide in lower scores in premature infants in the subdomains of verbal comprehension, perceptual reasoning, working memory and processing speed. Therefore, the conclusions that can be drawn in this regard are not very different from those provided by general IQ analyses. In any case, we would like to contribute to the discussion with a hypothesis proposed by Brydges et al. (2018). They propose that, while the differences in IQ between premature and full-term infants may be striking, differences concerning specific executive functions and intelligence skills may also be remarkable. General cognitive ability develops during early childhood, while specific executive functions and intelligence skills only begin to mature in middle or late childhood (about 10 years old). Therefore, the association between GA and cognitive functioning at a general level may be perceived from an early age. In contrast, specific abilities (which begin to develop in older children) are not affected by GA or birth weight. In our case, we could not test this hypothesis; on the one hand, there are few studies in this review that made such differentiation between general and specific IQ and, on the other hand, the review includes studies with samples aged 6–12 years, when the development of specific executive functions usually occurs around the age of 10 years.

However, at the individual level, some variability in performance occurs. Although it was not the main objective of this review, we could organize the information from these studies regarding possible risk factors for the cognitive development of these children. There are many associated factors, both medical/perinatal factors (GA, birthweight, brain injury) and family/social factors (parental education, socioeconomic status).

Researchers are progressively incorporating person-centered approaches to identify clusters of children with similar cognitive patterns. Regarding biological factors, many works analyzed the association with GA. The results of these papers show that IQ scores were significantly lower in children born EPT and VPT compared to those born at term, but no differences were found in children born MLPT compared to full-term children. These results are consistent with those of other studies (Joseph et al., 2022). GA is strongly associated with intellectual development. The studies included in this review report that cognitive function is significantly worse the shorter the gestation (Hutchinson et al., 2013; Joseph et al., 2016; Cheong et al., 2017; Heeren et al., 2017; Domellöf et al., 2020; Kaul et al., 2021b; Carmo et al., 2022). The main hypothesis to explain this association is the greater immaturity at shorter GA. As indicated by Torres et al. (2016) in their review, the more severe alterations presented by extreme preterm infants may be related to the higher prevalence of severe medical complications (hypoxic-ischemic sequelae such as periventricular leukomalacia, intraventricular hemorrhage, or germinal matrix and periventricular infarcts, etc.) and are associated with brain abnormalities.

Although other areas have provided evidence of the role of both heredity and environment in child development, very few studies in this review have analyzed social or family variables in the case of premature infants, thus the conclusions in this regard are less corroborated by the studies. Among them, only the mother's education level appears repeatedly in some studies as a factor that is positively associated with children's cognitive functioning, and, to a lesser extent, negatively associated with socioeconomic disadvantages. In addition, although the dynamic role of these influences has been shown, the effect of social factors on cognitive development changes across childhood. Lower mother's education and lower socioeconomic status were not associated with cognitive outcomes at 2 years in a sample of preterm children, but were increasingly associated with poorer cognitive outcomes across childhood and adolescence (Doyle et al., 2015). Given that proximal sociofamiliar factors, such as parenting, are related to cognitive and academic outcomes in children born very preterm, more research is recommended to learn about the influence of these variables over time and how they may interact with other factors. An example of this interaction is shown by Bilsteen et al. (2021). Their findings corroborate that shorter GA and lower parents' education level are associated with poorer school outcomes at the age of 16 years, but also that parents' education level mitigates the adverse effects of shorter GA on school outcomes.

As limitations of this work, it should be pointed out that the possible effect of the interventions was not included as a criterion in the review protocol. For that reason, no articles were found in the search that considered the influence of interventions on preterm IQ. In this regard, other investigations show that early intervention improves the cognitive outcomes of premature infants (Nordhov et al., 2010). Likewise, there is a wide variety of interventions in the family of preterm children that reduce parental stress (Martínez-Shaw and Sánchez-Sandoval, 2022). New intervention programs with preterm newborns developed in recent decades, such as the Developmental Centered Care Model (CCD) or the Newborn Individualized Developmental Care and Assessment Program (NIDCAP), were not considered. Another limitation is the fact that, as most of the articles are from developed countries, it is difficult to generalize the results to the world population. Further studies should be conducted in developing countries.

As future lines of intervention, it would be interesting to conduct a meta-analysis that, although it may include a smaller number of articles or variables to be studied, would quantitatively update the cognitive outcomes of school-aged premature children. Likewise, it would be appropriate to incorporate subsamples with clinical populations in future studies, which would allow examining the cognitive development of children with several pathologies associated with prematurity.

## Data availability statement

The original contributions presented in the study are included in the article/supplementary material, further inquiries can be directed to the corresponding author.

## Author contributions

YS-S designed the study. LL and YS-S wrote the manuscript. All authors collaborated in data extraction and coding and contributed to manuscript revision.
